# Mu Opioid Receptor Heterodimers Emerge as Novel Therapeutic Targets: Recent Progress and Future Perspective

**DOI:** 10.3389/fphar.2020.01078

**Published:** 2020-07-15

**Authors:** Li Zhang, Jiang-Tao Zhang, Lihua Hang, Tong Liu

**Affiliations:** ^1^ Department of Anesthesiology, The First People's Hospital of Kunshan Affiliated with Jiangsu University, Kunshan, China; ^2^ Jiangsu Key Laboratory of Neuropsychiatric Diseases and Institute of Neuroscience, Soochow University, Suzhou, China; ^3^ College of Life Sciences, Yanan University, Yanan, China

**Keywords:** pain, opioid, opioid receptor, heterodimer, side effect

## Abstract

Opioids are the most effective analgesics used in the clinical management of cancer pain or non-cancer pain. However, chronic opioids therapy can cause many side effects including respiratory depression, nausea, sedation, itch, constipation, analgesic tolerance, hyperalgesia, high addictive potential, and abuse liability. Opioids exert their effects through binding to the opioid receptors belonging to the G-protein coupled receptors (GPCRs) family, including mu opioid receptor (MOR), delta opioid receptor (DOR), and kappa opioid receptor (KOR). Among them, MOR is essential for opioid-induced analgesia and also responsible for adverse effects of opioids. Importantly, MOR can form heterodimers with other opioid receptors and non-opioid receptors *in vitro* and *in vivo*, and has distinct pharmacological properties, different binding affinities for ligands, downstream signaling, and receptor trafficking. This mini review summarized recent progress on the function of Mu opioid receptor heterodimers, and we proposed that targeting mu opioid receptor heterodimers may represent an opportunity to develop new therapeutics, especially for chronic pain treatment.

## Introduction

Chronic pain is a distressing and debilitating disease, which affects one-third of the population worldwide (Basbaum et al., 2009). Opioids such as morphine, codeine, hydrocodone, oxycodone, fentanyl, and tramadol, are considered the most effective analgesics used in the clinical management of chronic pain, which are among the most commonly prescribed and frequently abused drugs in the US (Ballantyne and Mao, 2003; Dart et al., 2015; Skolnick, 2018). However, opioids also cause many side effects associated with their acute use (including respiratory depression, nausea, sedation, itch, and constipation) and prolonged use (analgesic tolerance, hyperalgesia, high addictive potential and abuse liability) (Ballantyne and Mao, 2003; Schuckit, 2016). Rewarding and euphoric properties of opioids significantly contribute to their abuse potential (Darcq and Kieffer, 2018). Opioid tolerance is defined as a reduction in effect following prolonged drug administration that results in a loss of drug potency, resulting in the need to increase the opioids dosage to maintain the initial effects (Mao et al., 1995). Opioid-induced hyperalgesia (OIH) refers to the development of hypersensitivity to painful stimuli during chronic opioids administration (Mao et al., 1995; Roeckel et al., 2016). Opioid tolerance and OIH are considered to significantly contribute to opioid epidemic worldwide (Skolnick, 2018). These adverse effects of opioid dramatically reduce the quality of life of those patients with chronic pain (Ballantyne and Mao, 2003; Volkow and McLellan, 2016). On the other hand, opioid abuse and opioid overdoses in the US exceed 45,000 deaths per annum, representing the major cause of accidental deaths (Dart et al., 2015; Blendon and Benson, 2018). Although opioid receptor antagonists (such as naltrexone and naloxone) can lessen addictive impulses and facilitate recovery from opioid overdose, opioid antagonists also have severe side effects due to the disruption of endogenous opioid system (Boyer, 2012). Since misuse and/or abuse prescribed opioid drugs reach epidemic levels, designing new effective opioid analgesics without side effects may provide an important strategy against this opioid crisis (Blendon and Benson, 2018).

## Overview of Opioid Receptors Signaling

**Figure 1:**
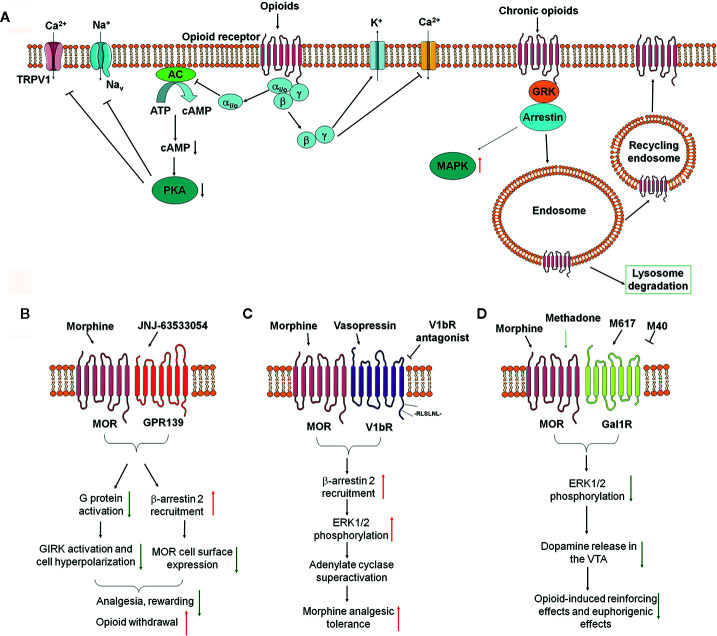
The impact of opioid receptor heterodimerization on opioid receptor signaling, trafficking, and behavioral outcomes. **(A)** Classical opioid receptor signaling pathways. **(B–D)** Recent identified opioid receptor heterodimers and downstream signaling in the nervous system and their effects on opioid side effects, including MOR-GPR139 **(B)**, MOR-V1bR **(C)**, and MOR-GalR1 **(D)**.

Opioids exert their effects through binding to the opioid receptors belonging to the G-protein coupled receptors (GPCRs) family, including μ-opioid receptor (MOR), δ-opioid receptor (DOR), and κ-opioid receptor (KOR) (Stein, 2016). After binding of a ligand, opioid receptors activate intracellular pertussis toxin-sensitive heterotrimeric G_i/o_ protein to dissociate into G_αi/o_ and G_βγ_ subunits, which initiate downstream signaling. G_αi/o_ inhibits adenylyl cyclases and cAMP production, and protein kinase A (PKA), resulting in modulating ion channels in the membrane, such as inhibition of transient receptor potential cation channel subfamily V member 1 (TRPV1) and voltage-gated sodium channels (VGSCs) (Machelska and Celik, 2018). G_βγ_ blocks calcium channels and opening of potassium channels, including G protein-coupled inwardly rectifying potassium (GIRK) channels and adenosine triphosphate-sensitive potassium channels (K_ATP_) (**Figure 1A**). Opioids attenuate neuronal excitability and excitatory neurotransmitters release from presynaptic terminals, which contribute to opioid-induced analgesia. Opioid receptors can be phosphorylated by GPCR kinases (GRKs) to promote the binding of β-arrestins, leading to opioid receptors desensitization and receptor internalization through clathrin-dependent pathways, resulting in reduced cell surface expression (Darcq and Kieffer, 2018). Dephosphorylated opioid receptors can then be recycled to the plasma membrane, or targeted into lysosome for degradation (**Figure 1A**).

Opioid receptors are expressed by many types of cells, including neurons in the central and peripheral nervous system, neuroendocrine cells, immune cells, and ectodermal cells (Stein, 2016; Machelska and Celik, 2020). Thus, opioid receptors are required and are driver for the production of many adverse effects following prolonged opioid therapy (Darcq and Kieffer, 2018; Machelska and Celik, 2018). For example, MOR is essential for opioid-induced analgesia and also responsible for many adverse effects of opioids, including tolerance, hyperalgesia, respiratory depression, constipation, nausea, and reward/euphoria that may lead to addiction (Darcq and Kieffer, 2018; Kibaly et al., 2019; Zhang et al., 2020). In addition, chronic opioid treatment may cause complex maladaptive responses, including downregulation of the opioid receptors, activation of anti-opioid systems, altering neuronal circuitry, activation of glial cells (including microglia and astrocytes), and even gut microbiota dysbiosis (Hutchinson et al., 2011; Williams et al., 2013; Guo et al., 2019).

## Mu Opioid Receptor Heterodimers *In Vitro* and *In Vivo*

Many family-A GPCRs are found to be able to form receptor heterodimers, including opioid receptors (Ferré et al., 2014; Ferré, 2015; Gomes et al., 2016). Heterodimers represent another important layer of functional complexity of opioid receptors and provide additional opportunity for modulating the function of opioid receptors (Machelska and Celik, 2018). Opioid receptor heterodimers can form between opioid receptors (Li-Wei et al., 2002) or between opioid receptors and non-opioid receptors (Jordan and Devi, 1999; Costantino et al., 2012). For example, it has been revealed that opioid receptor heterodimers exist *in vitro* and *in vivo*, including MOR-MOR homodimers (Li-Wei et al., 2002), MOR-DOR heterodimers (Costantino et al., 2012), MOR-KOR heterodimers (Chakrabarti et al., 2010), and DOR-KOR heterodimer (Jordan and Devi, 1999), MOR1D-Gastrin Releasing Peptide Receptor (GRPR) heterodimer (Liu X. Y. et al., 2011), MOR-cholecystokinin B receptor (CCKBR) heterodimer (Tao et al., 2017), MOR-cannabinoid 1(CB1) heterodimer (Rios et al., 2006), MOR-chemokine receptor 5(CCR5) heterodimer (Suzuki et al., 2002), MOR-α_2A_ adrenergic receptor (α_2A_AR) heterodimer (Vilardaga et al., 2008), MOR-dopamine D1 heterodimer (Tao et al., 2017), and MOR-nociceptin receptor heterodimer (Costantino et al., 2012). The formation of different types of opioid receptor heterodimers through allosteric mechanisms over the receptor interface may alter pharmacological properties of opioid ligands and may produce additional pharmacological subtypes. In addition, opioid receptor heterodimers may also have dramatic impact on opioid receptor trafficking and downstream signaling. Given opioid receptor heterodimers are often expressed on specific and limited brain regions and involved in many adverse effects induced by chronic opioid therapy, targeting opioid receptor heterodimers and subsequent downstream signaling may represent a novel therapeutic strategy for the treatment of chronic pain and adverse effects of opioids.

## Identification, Downstream Signaling, and Function of Mu Opioid Receptor Heterodimers

Accumulating evidence demonstrated that mu opioid receptor can form heterodimers with other opioid receptors or non-opioid receptors *in vitro* and *in vivo*, which have distinct pharmacological properties, different binding affinities for ligands, distinct downstream signaling, and trafficking (Jordan and Devi, 1999; Gomes et al., 2011; Ugur et al., 2018). In this mini review article, we highlighted the recent identified mu opioid receptor heterodimers, their pharmacological properties, and downstream signaling, and their involvement in pathological conditions (**Table 1**). We finally discussed the potential therapeutic potentials by targeting these opioid receptor heterodimers, especial for chronic pain management.

**Table 1:** Identification, downstream signaling, and function of Mu opioid receptor heterodimers *in vitro* and *in vivo*.

Receptor pair	Tissue distribution	Methods	Ligand binding, downstream signaling and trafficking	Behavioral outcomes	References
MOR-DOR	Mouse brain, SC, DRG	Co-IP, disruptive peptides	Positive binding cooperativity, increased G protein signaling	ND	(Erbs et al., 2015)
	Rat NRM	Whole-cell recording, behavioral test	Synergy upon co-activation of MOR and DOR	Increased analgesia	(Zhang and Pan, 2010)
	Rat RVM	Behavioral test	Synergy upon co-activation of MOR and DOR	Increased analgesia	(Sykes et al., 2007)
	DRG	Electrophysiological recording	Inhibitory coupling of MOR to VDCCs	ND	(Walwyn et al., 2009)
	DRG	Co-IP, disruptive peptides	DOR phosphorylation at Thr-161 is required for the formation of DOR-MOR heterodimers	Morphine analgesic tolerance	(Xie et al., 2009)
	DRG, SC	Co-IP, disruptive peptides	DOR activation led to endocytosis and degradation of surface MOR	Disrupted MOR-DOR enhanced morphine analgesia and reduced morphine tolerance	(He et al., 2011)
	SC	Co-IP, BRET	DOR antagonists enhance MOR binding and signaling activity	DOR antagonists enhance morphine analgesia	(Gomes et al., 2004)
	brain	Antibody to MOR-DOR heterodimers, heterodimer biased ligand (CYM51010)	MOR-DOR co-internalization	CYM51010 induced analgesia similar to morphine, but less analgesic tolerance	(Gomes et al., 2013)
	Striatum, hippocampus	Deltorphin-II	Gαz activation, and no uncoupling after chronic morphine	ND	(Kabli et al., 2014)
	DRG	Co-localization, Deltorphin-II	DAMGO induced DOP receptor internalization and trafficking following chronic morphine	ND	(Ong et al., 2015)
	Brain	Antibody to MOR-DOR heterodimers	Increased MOR-DOR abundance following chronic morphine	ND	(Gupta et al., 2010)
	Brain, DRG, SC	Co-IP, redMOR/greenDOR double knock-in mice	MOR and DOR neuronal co-expression in dorsal root ganglia, spinal cord, hippocampus, LH, basal nucleus of Meynert, and piriform cortex.	ND	(Scherrer et al., 2009)
	Brain, SC	DORGFP mice and MORmCherry mice	DOR and MOR is limited to small populations of the spinal cord and is rare in parabrachial, amygdalar, and cortical brain regions related to pain.	ND	(Wang et al., 2018)
	DRG	ISH, single-cell PCR, immunostaining	Coexistence of DORs and MORs in small DRG neurons; Both DOR and MOR agonists reduce Ca^2+^ currents in DRG neurons and inhibit C-fiber synaptic transmission in the spinal cord.	ND	(Wang et al., 2010)
MOR-KOR	Rat SC in female	Co-IP, cross-linking experiments	Synergy upon co-activation of MOR and KOR	Spinal morphine analgesia in female	(Chakrabarti et al., 2010)
MOR-CB1	Rat striatum	Electron microscopic immunocytochemical labeling	MOR and CB1 are partially colocalized in dendrites in striatum	ND	(Rodriguez et al., 2001)
	Striatum	BRET	Reciprocal cross antagonism	Co-activation of MOR and CB1 receptors leads to a attenuation of the response upon activation of individual receptors for neuritogenesis	(Rios et al., 2006)
	Rat or mouse NAcC	Behavioral, pharmacological, electrophysiological methods	Bidirectional negative crosstalk	Co-activation of MOR and CB1 receptors increased social play behaviors	(Manduca et al., 2016)
MOR-Gal1R	Mouse VTA	BRET, BiFC, disrupting synthetic peptide	Cross-antagonistic interactions between MOR and Gal1R	Opioids-induced reward	(Moreno et al., 2017)
	Mouse VTA	BRET, microdialysis, [^18^F]FDG PET imaging.	MOR-Gal1R attenuates the potency of methadone, but not other opioids, in stimulating the dopamine release in the VTA	Opioids-induced euphoria	(Cai et al., 2019)
MOR-alpha2A	Hippocampal neurons	Co-IP, BRET,	Activation of either MOR or alpha2A receptors leads to an increase in the extent of signaling, whereas activation of both receptors leads to a decrease.	ND	(Tan et al., 2009)
	Rat NTS	Co-IP, In situ proximity ligation assays, immunofluorescence staining	MOR-alpha2A reduced the NO-dependent depressor effects of activation of alpha2A receptor.	Hypertension	(Sun et al., 2015)
		FRET	Cross-inhibition upon agonist coactivation	ND	(Vilardaga et al., 2008)
MOR-GPR139	Mouse brain, including VTA, PAG, CPu, and DRG	ISH, co-IP,	Increased β-arrestin signaling, inhibition of G protein signaling, impede MOR trafficking to the cell surface	Diminished morphine analgesia, suppressed morphine self-administration	(Wang et al., 2019)
MOR-CCR5	Human and monkey lymphocytes	Co-IP, chemical crosslinking experiments	Combination treatment of cells with morphine, an agonist for mu, and MIP-1beta, a ligand for CCR5, suppresses the inhibitory effect of MIP-1beta and increases the stimulatory effect of morphine on CCR5 expression.	ND	(Suzuki et al., 2002)
	Rat PAG	Behavioral tests	Activation of CCR5 led to desensitization of MOR	Activation of MOR-CCR5 increased nociception	(Szabo et al., 2002)
MOR-CCKBR	Rat SC and DRG	Co-IP, FLIM-FRET, MOR mutant construction, cell-penetrating interfering peptide	Weakened the activity of MOR	CCK-8 antagonism to morphine analgesia in rats	(Yang et al., 2018)
MOR-V1bR	Mouse RVM	ISH, co-IP, BRET, Truncated V1bR receptor, genome editing	Increased β-arrestin-2 signaling, upregulation of ERK phosphorylation and adenylate cyclase sensitization	Morphine analgesic tolerance	(Koshimizu et al., 2018)
MOR1D-GRPR	Mouse SC	Co-IP, disruptive peptide	Cross-activate GRPR signaling, including PLCβ3 and IP3R3 signaling.	I.t. injection of morphine-induced itch in mice	(Liu X. Y. et al., 2011)

BiFC, Bimolecular fluorescence complementation; BRET, bioluminescence resonance energy transfer; CCKBR, cholecystokinin type B receptor; CCR5, C-C chemokine receptor type 5; Co-IP, Co-immunoprecipitation; CPu, Caudate Putamen; DAMGO, [D-Ala^2^, N-MePhe^4^, Gly-ol]-enkephalin; DRG, Dorsal Root Ganglion; FLIM-FRET, fluorescence lifetime-imaging-microscopy-based fluorescence resonance energy transfer; Gal1R, galanin-1 receptor; ISH, in situ hybridization; LH, lateral hypothalamus; NAcC, nucleus accumbens core; ND, not determined; NRM, nucleus raphe magnus; NTS, nucleus tractus solitarii; PAG, Periaqueductal Gray; PET, positron emission tomography; RVM, rostral ventromedial medulla; V1bR, vasopressin 1b receptors; VDCCs, voltage-dependent calcium channels; VTA, Ventral Tegmental Area.

## MOR-DOR Heterodimer

To date, among all MOR heterodimers, MOR-DOR heterodimer is the most importantly known and proven to exist both *in vitro* and *in vivo*. Several agonists and antagonists selective for MOR-DOR heterodimer have been identified and synthesized (Olson et al., 2018). George et al. reported that the MOR and DOR may form heterodimer when they were co-expressed *in vitro*, and selective agonists for each receptor had reduced potency (George et al., 2000; Gomes et al., 2013). By using co-immunoprecipitation (co-IP), bioluminescence resonance energy transfer (BRET), and fluorescence resonance energy transfer (FRET), it was demonstrated that physical interactions between MOR and DOR upon co-expression *in vitro* (Gomes et al., 2004; Yekkirala et al., 2012). It was reported that MOR agonist DAMGO activates Gαi/o-mediated signaling in MOR-expressing cells while activates β-arrestin 2 for changing the dynamics of ERK-mediated signaling in MOR-DOR heterodimer-expressing cells (Rozenfeld and Devi, 2007). DOR selective agonist SNC80 induced intracellular calcium release only in MOR-DOR heterodimers expressing cells by using a chimeric G-protein-mediated calcium fluorescence assay (Metcalf et al., 2012). CYM51010, a MOR-DOR heterodimer selective agonist, induced recruitment of β-arrestin 2 and GTPγS binding, which was blocked by a MOR-DOR heterodimer selective antibody (Gomes et al., 2013). The delta opioid receptor (DOR) exerts an antagonistic allosteric influence on MOR function within a MOR-DOR heterodimer. DOR antagonist enhances MOR recognition, G_i/o_ coupling and inhibition of cAMP levels (Gomes et al., 2004). Thus, these results suggested that MOR-DOR heterodimer affected the pharmacological properties of each receptor.

MOR-DOR heterodimer may also have distinct intracellular trafficking. Previous study showed that MOR and DOR can form heterodimers only when they are present at plasma membrane (Milan-Lobo and Whistler, 2011). In contrast, another study showed that MOR-DOR heterodimers located in the endoplasmic reticulum, where they recruit ${\text{G}}_{{\text{α}}_{\text{z}}}$ protein (Hasbi et al., 2007). Some agonists (e.g. DAMGO, Deltorphin II, SNC80, and methadone) can induce MOR-DOR heterodimer endocytosis, but others (e.g. DADPE) cannot do so (Décaillot et al., 2008). In addition, RTP4, a Golgi chaperone, was identified as a key regulator of the levels of MOR-DOR heterodimers at the cell surface (Décaillot et al., 2008). It was also found that treatment with DOR agonists led to endocytosis of both DORs and MORs, further processed for degradation, resulting in a reduction of surface expression of MOR (He et al., 2011). These effects were attenuated by treatment with an interfering peptide that disrupted the MOR-DOR heterodimer formation (He et al., 2011).

MOR-DOR heterodimers are also demonstrated to distribute in central nervous system and play key roles in the regulation of pain and opioid analgesia. RedMOR/greenDOR double knock-in mice were generated to perform dual receptor mapping throughout the whole brain (Erbs et al., 2015). In the forebrain, MOR and DOR are mainly detected in separate population of neurons. In contrast, neuronal co-localization is detected in subcortical networks, which is essential for eating, sexual behaviors or response to aversive stimuli (Erbs et al., 2015). Recently, it was found that MOR and DOR co-expression is limited to small populations of neurons in the spinal cord. MOR and DOR co-expression is rare in parabrachial, amygdala, and cortical brain regions for pain processing (Wang et al., 2018). By using antibodies selective for MOR-DOR heterodimers generated by subtractive immunization strategy, it was found that up-regulation of MOR-DOR heterodimers were induced by chronic morphine treatment (Gupta et al., 2010). Tail-flick assay showed that CYM51010 targeting the MOR-DOR heterodimer demonstrated analgesic activity comparable to morphine, and chronic administration of CYM51010 resulted in less analgesic tolerance (Gomes et al., 2013). Recently, it was found that MOR-DOR heterodimer expression was up-regulated in injured dorsal root ganglia neurons and subcutaneous injection of CYM51010 inhibited neuropathic pain in rats and mice (Tiwari et al., 2020). Intriguingly, the CYM51010-induced analgesia still persisted in morphine-tolerant rats and was abolished in MOR knockout mice (Tiwari et al., 2020). In addition, intrathecal administration of a peptide to disrupt the formation of MOR-DOR heterodimers can enhance morphine analgesia and reduce analgesic tolerance in mice (He et al., 2011). Together, these results indicated that targeting MOR-DOR heterodimers may be a promising strategy to treat chronic pain or to improve therapeutic effects of opioids.

## MOR-KOR Heterodimers

Chakrabarti et al. (2010) found that the expression of MOR-KOR heterodimer was more prevalent in the spinal cord of proestrous vs. diestrous females and vs. males. Spinal morphine anti-nociception in females, but not males, required the concomitant activation of MOR and KOR in the spinal cord. Dynorphine was identified as a ligand for female-specific KOR within the MOR-KOR heterodimer. Co-IP experiments obtained with anti-KOR antibodies on the spinal cord showed that it is elevated in proestrus with high estrogen receptor (ER) levels (Liu N. J. et al., 2011). The gender- and ovarian steroid-dependent recruitment of MOR-KOR heterodimers may provide a way to influence the balance between anti-nociceptive and pro-nociceptive functions of the dynorphin/KOR opioid system in the spinal cord. Impaired formation of MOR-KOR heterodimers could be a biological determinant of various types of chronic pain states that are substantially more common in women than men. Thus, MOR-KOR may be therapeutic target for pain control, especial in women.

## MOR-GPR139 Heterodimers

Recently, Wang et al. (2019) identified a conserved orphan GPR139 receptor as a novel partner to form MOR-GPR139 heterodimers to negative regulate opioid receptor function and demonstrated that GPR139 can regulate opioid receptor signaling and trafficking. Firstly, Wang et al. (2019) established an ingenious forward genetic platform by using a model organism, *C. elegans* to screen mutations that affect opioid receptor function and signaling. Following the expression of mammalian MOR in the nervous system of nematodes (tgMOR), administration of morphine and fentanyl decreased locomotion in tgMOR nematodes. Based on the drug sensitivity of behavioral responses, they conducted a large-scale forward genetic screening of the transgenic nematode, and 900 mutants with altered opioid sensitivity were observed. By whole genome sequencing of these mutants, they identified an orphan receptor FRPR-13 (its homologous protein GPR139 in mammals) as a negative regulator of MOR function *in vivo*.

Wang et al. (2019) further investigated the functional interaction of MOR and GPR139 in transfected HEK cells. In transfected HEK293T cells, MOR activation causes hyperpolarization of membrane potential due to opening of the G protein-coupled inwardly-rectifying potassium channels (GIRKs) and expression of GPR139 inhibited these abovementioned effects. They showed that GPR139 and MOR can be co-immunoprecipitated in artificially overexpressed in a cellular context. When GPR139 is overexpressed at high amounts, they found that cell surface expressed MOR was reduced, suggesting that GPR139 may regulate MOR trafficking to the cell surface or its internalization. *In vitro*, GPR139 was further found to bind directly to MOR, promote the recruitment of β-arrestin 2, and inhibited the activation of G protein and GIRK channel. *In situ* hybridization experiments showed that GPR139 and MOR are expressed in the same brain regions, including the medial habenula (MHb) and locus coeruleus (LC). Wang et al. (2019) further provided electrophysiological evidence to support the functional interaction of MOR and GPR139. In cultured brain slices, GPR139 deficiency reduced the basal firing rate of MHb neurons and increased opioid sensitivity of LC neurons. Thus, it was concluded that GPR139 regulates the function of MOR in a cell-autonomous manner.

Wang et al. (2019) finally investigated the role of GPR139 in morphine-induced behavioral changes in mice. They observed that GPR139 knockout (KO) mice had normal baseline learning, nociception, locomotor activities, and motor coordination. However, GPR139 KO mice showed enhanced sensitivity to morphine-induced analgesia and reward effects. Administration of GPR139 agonists JNJ-63533054 inhibited morphine-induced analgesia and morphine-induced reward in mice. GPR139 KO mice also showed significantly diminished opioid withdrawal reactions (**Figure 1B**). Thus, these data indicate that inhibiting the function of GPR139 enhanced sensitivity to morphine-induced analgesia and reward effects, but diminished morphine withdrawal, while facilitating the function of GPR139 diminished morphine analgesia and rewarding effects. However, the formation and function of MOR-GPR139 heterodimers were only investigated *in vitro*. The precise localization and function of MOR-GPR139 heterodimers *in vivo* warrant further validation and investigation. Given GPR139 was identified as a novel anti-opioid system, brain regions selective targeting GPR139 or MOR-GPR139 heterodimers may be useful to improve the safety and efficacy of opioids.

## MOR-V1bR Heterodimers

Recently, Koshimizu et al. (2018) identified vasopressin 1b receptor (V1bR) as another partner to form MOR-V1bR heterodimers in promoting AC superactivation and the development of morphine tolerance, which is another excellent example that opioid receptor heterodimers may alter the opioid receptor signaling and function. Arginine-vasopressin (AVP), structurally related to oxytocin, are known to regulate morphine sensitivity and tolerance. However, the underlying mechanisms are poorly understood. Koshimizu et al. (2018) first found that V1bR knockout (KO) mice showed enhanced nociceptive thresholds and greater morphine sensitivity. In addition, the development of morphine tolerance was significantly delayed in V1bR KO mice. Next, they found that administration of selective V1bR antagonist SSR149415 (but not V1aR antagonist) into lateral ventricle of mice also reduced the development of morphine tolerance in mice. By using *in situ* hybridization analysis, they subsequently found that V1bR and MOR are co-localized in the rostral ventromedial medulla (RVM). They further examined the functional interaction between V1bR and MOR by using HEK cell co-expressing V1bR and MOR. In a single cell, bioluminescence resonance energy transfer (BRET) analysis confirmed that V1bR and MOR were located in close proximity (<10 nm). Binding experiments indicate that morphine binding to the MOR is substantially influenced by forming MOR-V1bR heterodimer in the cellular environment. AVP enhanced morphine-induced AC superactivation in the setting of V1bR-MOR heterodimers, which depends on β-arrestin 2 and extracellular regulated kinase (ERK) phosphorylation pathways. A leucine-rich segment in the C-terminal tail of the V1bR is essential for association with β-arrestin 2. Finally, they showed that deletion of the leucine-rich segment in the V1bR C-terminal tail by genome editing increased morphine analgesia and reduced AVP-mediated AC superactivation (**Figure 1C**). Altogether, it was proposed that targeting MOR-V1bR may be a novel approach for potentiating morphine analgesia with delayed development of morphine tolerance.

## MOR-Galanin Receptor 1 (Gal1R) Heterodimers

Previous studies showed that a neuropeptide galanin counteracts the behavioral effects of MOR agonists *in vivo*. Moreno et al. (2017) identified the formation of MOR-Gal1R heterodimer in transfected cells and in neurons in rat ventral tegmental area (VTA). MOR-Gal1R heterodimer plays a key role in the function of dopaminergic neurons and mediates the antagonistic interactions between MOR and Gal1R selective ligands (Moreno et al., 2017). Cai et al. (2019) further found an agonist-dependent function of MOR-Gal1R heterodimers and elucidated the mechanisms underlying opioid-induced rewarding, suggesting the formation of opioid receptor heterodimers may alter the pharmacological properties of opioid receptor. It was showed that MOR-GalR1 heterodimers in the rat VTA reduced the potency of methadone, but not other opioids (e.g. morphine and fentanyl), for stimulating dopamine release and producing euphoria (**Figure 1D**). Thus, these pharmacodynamic differences between methadone and morphine may provide a way to dissociate the euphoric versus therapeutic effects of methadone-like compounds. Consistently, patients on methadone maintenance experienced fewer euphoria compared with those using other opioids (Cai et al., 2019). Thus, these data indicated MOR-GalR1 heterodimers mediate the dopaminergic effects of opioids and novel methadone-like compounds with reduced potency to activate MOR-GalR1 heterodimers may be safer than current opioids. Given the critical role of MOR-GalR1 heterodimers in the determination of the potency of opioid-induced activation of rewarding pathways and restricted distribution of MOR-GalR1 heterodimers in the VTA, MOR-GalR1 heterodimers may be a promising target to develop safer opioid analgesics or even treat opioid addiction.

## MOR-CB1 Heterodimers

CB1 receptor is present in the peripheral and central nervous system, including primary sensory neurons in the DRGs, the spinal cord, and some brain regions related to pain processing (Bouchet and Ingram, 2020). Early study showed co-localization of CB1 and MOR in lamina II neurons in the spinal cord (Salio et al., 2001), and synergistic interactions also existed between cannabinoid and opioid analgesia (Raehal and Bohn, 2014). By using biophysical methods, Rios et al. demonstrated that CB1 can form heterodimers with MOR in transfected cells (Rios et al., 2006). Co-activation of MOR-CB1 leads to cross-inhibition of neurite outgrowth involving inhibition of the Src-STAT3 pathway, suggesting antagonistic allosteric interactions in CB1-MOR heterodimers (Rios et al., 2006). Thus, MOR-CB1 heterodimer may be a target to modulate neuronal plasticity.

## MOR-CCKBR Heterodimers

Previous studies found the antagonism of cholecystokinin octapeptide (CCK8) to opioid analgesia (Pu et al., 1994), and studies using L-365,260 (a specific antagonist of CCKBR) showed that CCK-8 inhibited opioid analgesia through CCKBR (Dourish et al., 1990). Recently, Yang et al. (2018) demonstrated that MOR and CCKBR could form heterodimer and MOR-CCKBR heterodimer may underlie the antagonism of CCK8 to opioid analgesia. They first validated the co-localization of MOR and CCKBR in neurons in spinal cord dorsal horn and the DRGs by using double-labeling immunofluorescence staining. By using co-IP and FLIM-FRET methods, they further validated that CCKBR and MOR form heterodimers in transfected HEK293 cells and the transmembrane domain 3 (TM3) domain of MOR play a key role in the formation of MOR-CCKBR heterodimers. Further, the formation of MOR-CCKBR heterodimer leads to decreased MOR affinity and reduced agonist-mediated phosphorylation of ERK1/2 in transfected HEK293 cells. They developed a cell-penetrating interfering peptide (TM3_MOR_-TAT) by adding the TAT sequence (RKKRRQRRR) to the C terminal of the entire TM3, in order to disrupt the formation of MOR-CCKBR heterodimer. Finally, they found that TM3_MOR_-TAT enhanced MOR signaling in transfected cells and prevented CCK8-induced antagonism against morphine analgesia in rats. Thus, MOR-CCKBR may be a promising therapeutic target for enhancing morphine analgesia.

## MOR-α2AAR Heterodimers

Previous study showed that there exists a conformational antagonistic crosstalk between α2AAR and MORs in their downstream signaling upon co-activation (Vilardaga et al., 2008). Yang et al. (2018) demonstrated that these receptors, either singly or as a heterodimer, activate common signal transduction pathways mediated through the inhibitory Gαi/o. Using FRET microscopy, they showed that within the MOR-α2AAR heterodimer, the MOR and α2AAR communicate with each other through a cross-conformational switch that permits direct inhibition of one receptor by the other (Vilardaga et al., 2008). It was also found that morphine binding to the MOR triggers a conformational change in the norepinephrine-occupied α2AAR that inhibits its signaling to G_αi_ and the downstream MAP kinases (Vilardaga et al., 2008). These data highlight a new mechanism in signal transduction whereby a G protein-coupled receptor heterodimer mediates conformational changes that propagate from one receptor to the other and cause the second receptor's rapid inactivation. Thus, these results suggest that activation of MOR-α2AAR heterodimers by combined agonists for each other could play a role in counteracting excessive analgesia.

However, early work indicates that combined agonists acting on α2AAR and opioid receptors have analgesic properties and act synergistically when co-administered in the spinal cord (Stone et al., 1997). Norepinephrine (NE) or clonidine (α2AAR agonists) significantly reduces the evoked release of glutamate from spinal cord synaptosomes (Kamisaki et al., 1993) and the release of substance P (SP) and calcitonin gene related peptides (CGRP) from spinal cord slices (Bourgoin et al., 1993). In addition, immunostaining data showed that both α2AAR and MOR was observed in the superficial layers of the dorsal horn of the spinal cord and the primary localization of the α2AAR in the rat spinal cord is on the terminals of capsaicin-sensitive, SP-containing primary afferent fibers (Bourgoin et al., 1993). Thus, although α2AAR and MOR may co-localized on these primary afferent fiber terminals, the formation of MOR-α2AAR heterodimer cannot explain the synergy of agonists of MOR and α2AAR in analgesia.

## MOR1D-GRPR Heterodimers

The mouse Oprm gene encodes 16 exons, comprising dozens of spliced isoforms that primarily differ at C terminus (Pasternak and Pan, 2013). Liu X. Y. et al. (2011) demonstrated one MOR isoform MOR1D is co-expressed with GRPR in superficial layers of the spinal cord in mice. Further, MOR1D was shown to be associated with GRPR to form MOR1D-GRPR heterodimer in the spinal cord (Liu X. Y. et al., 2011). Activation of MOR1D unidirectionally cross-activates GRPR and its downstream effectors (such as PLC3β and IP3R3) in neurons of spinal cord. Blocking the formation of MOR1D-GRPR by a disrupted peptide attenuated morphine-induced itch, but did not affect morphine-induced analgesia in mice (Liu X. Y. et al., 2011). Thus, the results indicated that MOR1D-GRPR may be a promising therapeutic target for treating morphine-induced itch, which is a remarkable side effect of morphine.

## Targeting Mu Opioid Receptor Heterodimers as Novel Therapeutic Strategies

In order to design new opioid analgesics without side effects, we need to better understand the molecular and cellular mechanisms underlying the regulation of MOR function. So far, it is proposed several different strategies to achieve this goal (Machelska and Celik, 2018). For example, one strategy is selective targeting MOR splice variants that are selective involved in analgesia. Another strategy is the biased activation of MOR towards analgesia-associated intracellular signaling pathways. Third strategy is selective targeting MOR at peripheral nervous system though reducing opioid access to the central nervous system. Fourth strategy is selective targeting MOR in peripheral inflamed tissue. Fifth strategy is identification of compounds that specific target opioid receptor heterodimers. Heterodimers are defined as a protein complex composed of two functional receptor units (protomers) and have different biochemical and pharmacological properties than individual units. This research highlight will introduce some recent progress about opioid receptor heterodimers and discussed the abovementioned fifth strategy to design new generation of opioid analgesics with few side effects.

So far, there are several innovation strategies to design new compounds for targeting opioid receptor heterodimers, aiming at improving analgesic and reducing side effects. First, bivalent ligands can be designed for targeting opioid receptor heterodimers, which may be designed through using a 21-22-atom spacer to connect MOR agonist and other receptor antagonist involved in heterodimers. Accordingly, for example, MMG22 was designed to target MOR-mGluR5 heterodimers, which consists oxymorphamine (MOR agonist) and metoxy-2-methyl-6-(phenylethynyl)-pyridine (mGluR5 antagonist) connected by a 22-atom spacer. Second, monovalent compounds were also developed to target opioid receptor heterodimers. For example, N-naphthoyl-β-naltrexamine (NNTA) with mixed KOR agonist/MOR antagonist was designed to target MOR-KOR heterodimers, which produced analgesia, little tolerance, and little withdrawal signs. Third, multifunctional ligands can also be designed to target opioid receptor heterodimers. For example, TY027 with MOR/DOR agonistic and NK1 receptor antagonistic activities produced analgesia, little tolerance, less reward, less withdrawal signs, no effects on gastrointestinal transit in preclinical animal models. Finally, combination of two compounds for targeting opioid receptor heterodimers is another choice. For example, combination of MOR agonist and V1bR antagonist (SSR149415) to target MOR-V1bR heterodimers produced analgesia with little tolerance. Additionally, combination of Gal1R agonist and MOR agonist may be used to reduce euphoric effects of opioids. Thus, bivalent, monovalent, and multifunctional ligands or combination of two drugs targeting opioid receptor heterodimers may represent next generation of pain killers with reduced side effects.

## Future Perspective

Although scientists have made great progress in promoting our understanding on the function of opioid receptor heterodimers, there are many questions that remain to be resolved. First, the existence and physiological/pathophysiological function of opioid receptor heterodimers *in vivo* remains largely unknown, including recent identified MOR-GPR139 heterodimers. Second, the studies on the structure and function of opioid receptor heterodimers is very challenging and more powerful research tools need to be developed. Of note, the current methods heavily rely on the heterologous expressing cells and antibodies, which often have specificity issues. Importantly, the application of x-ray crystallography and/or cryo-electron microscopy may be helpful to provide structural basis for the formation of opioid receptor heterodimers and the interaction between selective ligands and heterodimers. Additionally, the generation of mutant mouse expressing fluorescently tagged MOR and its partners within heterodimers will be useful to explore the distribution of opioid receptor heterodimers *in vivo*, avoiding the specificity issue of antibodies. Of course, to develop antibodies specific for opioid receptor heterodimers is another way. Third, the expression of opioid receptor heterodimers in an endogenous context may be highly dynamic. Fourth, opioid receptors may form heteromers with ion channels, such as NMDA receptor (Rodríguez-Muñoz et al., 2012) and TRPV1 receptor (Scherer et al., 2017), and these kind of heteromers may have distinct function in physiological and pathophysiological conditions. However, this topic is out of the scope of this review. Together, the expression and the interaction changes of opioid receptor heterodimers in many pathological conditions (including chronic opioid treatment and many pathological pain conditions) warrant further investigation. Finally, in order to identify the compounds with specific target opioid receptor heterodimers, the high-throughput screening assays have to be established.

## Conclusion

Opioid receptor heterodimers regulate the opioid receptor function at multiple levels, including pharmacodynamic of ligands, the receptor trafficking, and intracellular downstream signaling pathways. Impressively, the list of opioid receptor heterodimers is growing rapidly. There are also several innovation strategies or high-throughput screen platform to be developed in order to targeting opioid receptor heterodimers. Thus, the identification and characterization of MOR heterodimers may provide valuable therapeutic targets for chronic pain and improving opioid analgesics.

## Author Contributions

LZ and J-TZ performed literature search and prepared the draft. LH and TL wrote the manuscript. All authors contributed to the article and approved the submitted version.

## Funding

TL is supported by the grants from National Natural Science Foundation of China (81870874), Natural Science Foundation of Jiangsu Province, China (BK20170004 and 2015-JY-029), and Jiangsu Key Laboratory of Neuropsychiatric Diseases (BM2013003). This work was also financed by the Clinical and basic research of encephalopathy (KYC004) and the Social Development Special Fund of Kunshan (KS1931).

## Conflict of Interest

The authors declare that the research was conducted in the absence of any commercial or financial relationships that could be construed as a potential conflict of interest.
